# A silicate dynamo in the early Earth

**DOI:** 10.1038/s41467-020-14773-4

**Published:** 2020-02-25

**Authors:** Lars Stixrude, Roberto Scipioni, Michael P. Desjarlais

**Affiliations:** 1 Department of Earth, Planetary, and Space Sciences, University of California, Los Angeles, CA 90095 USA; 2 Department of Earth Sciences, University College London, London, WC1E 6BT UK; 3 Pulsed Power Sciences Center, Sandia National Laboratories, Albuquerque, NM 87185 USA

**Keywords:** Core processes, Geophysics, Mineralogy

## Abstract

The Earth’s magnetic field has operated for at least 3.4 billion years, yet how the ancient field was produced is still unknown. The core in the early Earth was surrounded by a molten silicate layer, a basal magma ocean that may have survived for more than one billion years. Here we use density functional theory-based molecular dynamics simulations to predict the electrical conductivity of silicate liquid at the conditions of the basal magma ocean: 100–140 GPa, and 4000–6000 K. We find that the electrical conductivity exceeds 10,000 S/m, more than 100 times that measured in silicate liquids at low pressure and temperature. The magnetic Reynolds number computed from our results exceeds the threshold for dynamo activity and the magnetic field strength is similar to that observed in the Archean paleomagnetic record. We therefore conclude that the Archean field was produced by the basal magma ocean.

## Abstract (web summary)

Cooling of the iron core in the early Earth may have been too slow to allow for the generation of a magnetic field. Based on quantum mechanical and geodynamical modelling approaches, the authors find that the electrical conductivity of silicate liquid at high pressure and temperature conditions could have been sufficient to generate a silicate dynamo and a magnetic field in the early Earth.

## Introduction

The Archean magnetic field may have been essential for preserving the early atmosphere and hydrosphere^1^. The process by which the field is produced today: a dynamo hosted in the Earth’s metallic iron-rich liquid outer core is thought to be difficult to sustain in the early Earth because the core could not cool sufficiently rapidly^2–4^. Alternative energy sources, including radioactive heat production in the core or exsolution of Mg from the cooling core, may not be sufficient to sustain an early dynamo.

Planetary dynamos in the solar system are hosted in a variety of materials including iron-rich liquid, such as in the Earth and other terrestrial planets, ionically conductive fluid ices, as in Uranus and Neptune, and metallic hydrogen as in Jupiter and Saturn^5^. A silicate dynamo is so far unknown. Magnetohydrodynamic simulations, laboratory experiments, and studies of planetary bodies that host magnetic fields show that dynamo activity requires a sufficiently large magnetic Reynolds number $R_{\mathrm{m}} = \mu _0vL\sigma\; > \; 40$, where *μ*_0_ is the magnetic susceptibility, *v* is the flow velocity, *L* is the depth of the layer, and *σ* is the electrical conductivity^6,7^. Using illustrative values, $R_{\mathrm{{m}}} = 40\left( {\mathrm{{v}}}/1\;{{\mathrm{{cm/s}}}} \right)\left( {L/300\;{\mathrm{km}}} \right)\left( {\sigma /10,000\;{\mathrm{S/m}}} \right)$. Therefore, for a silicate dynamo to operate the electrical conductivity must exceed 10,000 S/m, more than 100 times higher than the highest values measured in silicate liquids at low pressure and temperature^8,9^, although less than typical metallic conductivity (10^6^ S/m for the Earth’s outer core).

Here we perform first principles molecular dynamics simulations of a silicate liquid that closely approximates the composition of the bulk silicate Earth and includes the six most abundant oxide components; the size of our simulation is 1129 atoms (see Methods). The simulations and the computation of the electrical conductivity closely follow our previous work on the SiO_2_ and (Mg,Fe)O systems^10,11^ (see Methods). Briefly, we compute the electronic contribution to the electrical conductivity with the Kubo–Greenwood formula^12^ and the ionic conductivity from the electric current autocorrelation function^13^. In order to investigate the effect of magnetism on our results, we consider non-spin-polarized and spin-polarized results with the latter characterized by high-spin moments on all the iron atoms. We combine our predictions of electrical conductivity with a model of the thermal evolution of the basal magma ocean to estimate the magnetic Reynolds number and the strength of the magnetic field generated by the silicate dynamo. We find that the magnetic Reynolds number exceeds 40 and that the field strength is in excellent agreement with paleointensity measurements.

## Results

### Electrical conductivity

**Fig. 1:**
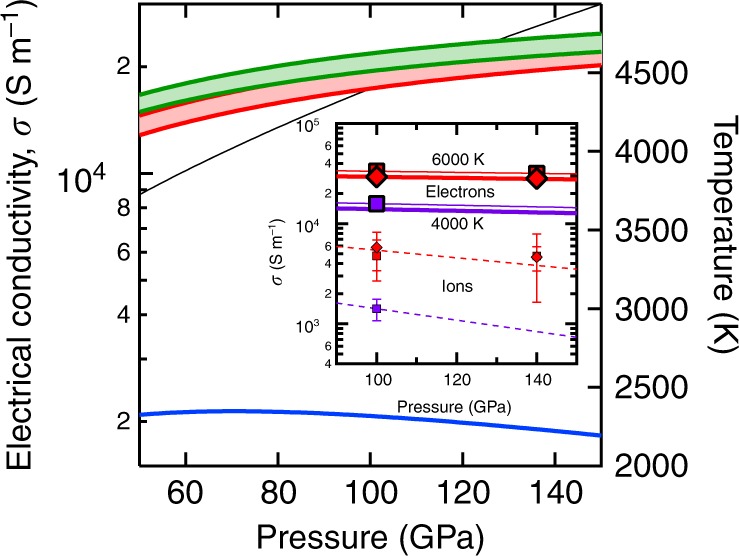
Electrical conductivity. The electronic *σ*_el_ (red), ionic *σ*_ion_ (blue), and total *σ*_total_ =  *σ*_el_ +  *σ*_ion_ (green) electrical conductivity along the magma ocean isentrope^37^ (black) for low-spin (top of range shown) and high-spin (bottom of range) results. Along the magma ocean isentrope, the electrical conductivity increases with depth because the effects of increasing temperature outweigh the effects of increasing pressure. Inset. The electrical conductivity of the bulk silicate Earth liquid from our simulations, including the electronic (large symbols) and ionic (small symbols with 1*σ* uncertanties) contributions at 6000 K (red) and 4000 K (purple) in spin-polarized (diamonds) and non-spin-polarized (squares) calculations. Lines are best fits to the form $\sigma = \sigma _0T^{ - 1}\exp \left[ { - \left( {E^\ast + PV^\ast } \right)/RT} \right]$ for the electronic non-spin-polarized results *(σ*_0_ = 1.994e9 S/m, E* = 108.6 kJ/mol, V* = 0.0611 cm^3^/mol, light solid lines) and shifted uniformly downward to account for spin-polarization *(σ*_0_ = 1.754e9 S/m, bold solid lines), and to the ionic contribution (*σ*_0_ = 1.0811e9 S/m, E* = 131.0 kJ/mol, V* = 0.437 cm^3^/mol, dashed lines).

Our results show that the electrical conductivity of silicate liquid exceeds 10,000 S/m at the conditions of the basal magma ocean (Fig. 1). The electrical conductivity increases with increasing temperature and decreases with increasing pressure. Magnetism reduces the electrical conductivity by 10%. The primary charge carriers are electrons: the electronic contribution to *σ* exceeds the ionic contribution by more than an order of magnitude. This behavior contrasts with experimental results at low pressure and temperature where ions are the dominant charge carriers^8^.

**Fig. 2:**
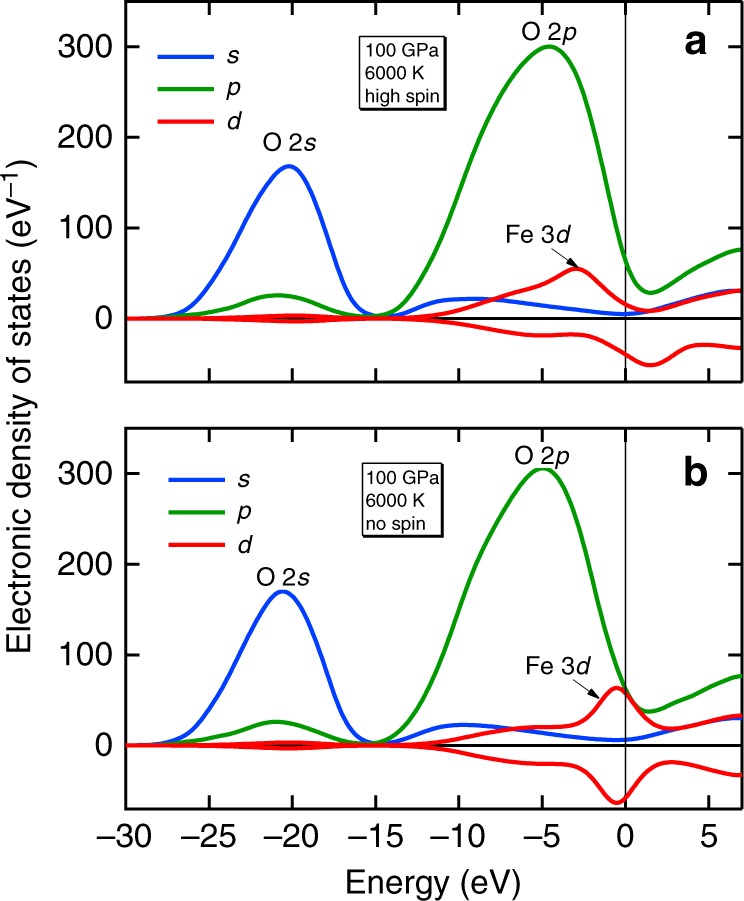
Electronic density of states in the silicate liquid. At 100 GPa and 6000 K in spin-polarized (**a**) and non-spin-polarized (**b**) states. Contributions from *s*- (blue), *p*- (green) and *d*-like (red) states are shown separately and the dominant atomic contributions to each are indicated. The vertical black line is the Fermi level.

We can understand the electrical conductivity of silicate liquid by comparing with simpler systems and by examining the electronic density of states (Fig. 2). The electrical conductivity of the silicate liquid is intermediate to that of SiO_2_ liquid^11,14,15^ and (Mg,Fe)O liquid^10^ over the range of temperature and pressure that we have investigated. The conductivity of the silicate liquid is greater than that of SiO_2_ because of the role of iron. Iron 3*d* states contribute to the density of states at the Fermi level and increase the conductivity compared with the iron-free system. The role of iron also explains why the temperature dependence of the electrical conductivity in the silicate liquid is less than that of SiO_2_ liquid (the conductivity in silica falls precipitously on cooling and drops ten times from 6000 to 4000 K, whereas in the silicate liquid, it decreases only twofold): the highly localized iron 3*d* states are less sensitive to their chemical environment than the O 2*p* states that also contribute to conduction and their contribution to the density of states at the Fermi level is much less sensitive to temperature. The conductivity of the silicate liquid is less than that of (Mg_0.75_Fe_0.25_)O because the iron concentration is less in the silicate liquid (Fe/(Fe + Mg) = 0.11). The electronic density of states also explains why the conductivity of the spin-polarized system is slightly less than that of the non-spin-polarized system: magnetism splits up- and down-spin iron 3*d* states, reducing the density of states at the Fermi level. While there are no experimental measurements of the electrical conductivity of silicate liquids at basal magma ocean conditions, we have shown that our approach yields results that are in good agreement with available experimental data at high pressure including the optical reflectivity of silica liquid and the electrical conductivity of crystalline (Mg,Fe)O^10,11^.

### Magnetic field

To quantify magnetic field production, we combine our results for the electrical conductivity with a model of the thermal evolution of the basal magma ocean. The thermal evolution model is identical to that examined previously^16^, except that we have replaced the previously assumed value of the entropy of melting with the value determined from first principles molecular dynamics simulations (see Methods). This model yields the thickness of the magma ocean *L* and heat flux out of the magma ocean *q*, which we combine with our values of the electrical conductivity to estimate the magnetic Reynolds number $R_{\mathrm{m}} = \left( {Lq/\rho H_{\mathrm{T}}} \right)^{1/3}L\mu _0\sigma$ where *ρ* is the mean density of the magma ocean and *H*_T_ is the thermal scale height. We have assumed the mixing length scaling for the flow velocity^17^. We estimate the magnetic field strength at the top of the basal magma ocean according to the scaling derived from numerical simulations^17^ $B_{\mathrm{M}} = 0.63\rho ^{1/3}\left( {Lq/H_{\mathrm{T}}} \right)^{2/3}$, where we have assumed that viscous dissipation is negligible because the ratio of viscous to magnetic diffusivity, the magnetic Prandtl number $P_m = \mu _0\sigma \eta /\rho = 10^{ - 7}$ is small, where *η* = 0.1 Pa s is the viscosity of the basal magma ocean from previous first principles simulations^18^. Finally, we estimate the field strength at the surface $B_{\mathrm{S}} = {\textstyle{1 \over 7}}B_{\mathrm{M}}\left( {r_{\mathrm{M}}/r_{\mathrm{E}}} \right)^3$, where the pre-factor accounts for the proportion of the field strength in the dipole component, and the cubic dependence on radius accounts for the upward continuation of the dipole component to the surface: *r*_M_ is the radius at the top of the basal magma ocean and *r*_E_ is the radius of the Earth^6^.

**Fig. 3:**
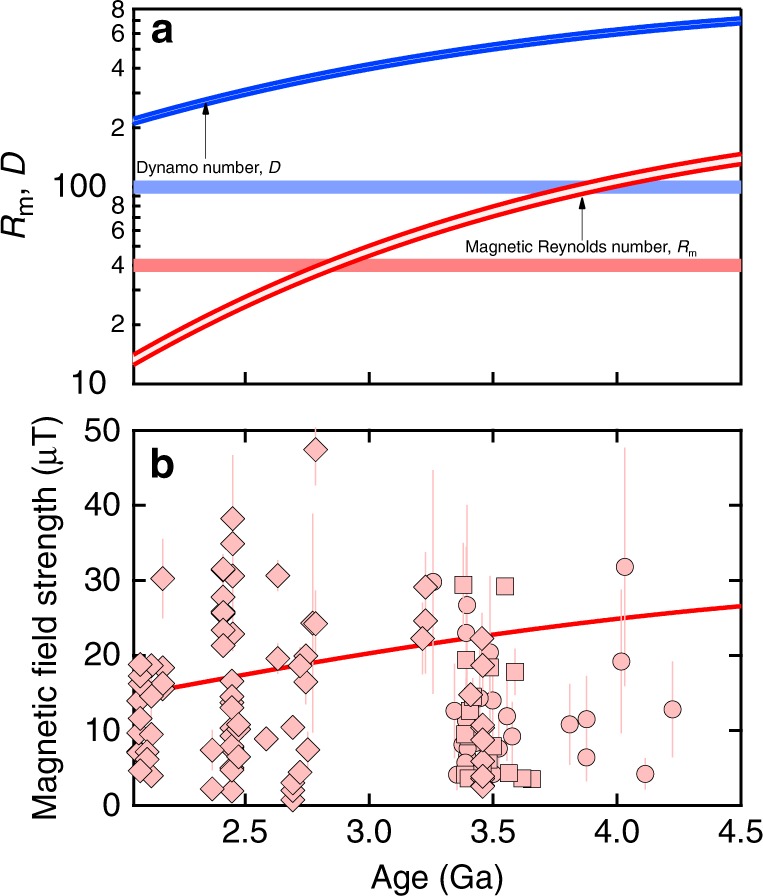
Magnetic Reynolds number and magnetic field strength. **a** The magnetic Reynolds number (red) and Dynamo number (blue) computed from our electrical conductivity results and thermal evolution model with shading indicating the difference between low-spin (top of range) and high-spin (bottom of range) results compared with the respective critical values of *R*_m_ = 40 and *D* = 100 (thick horizontal lines). **b** Magnetic field strength computed from our model (red line) compared with paleomagnetic data: diamonds from the PINT database^40^, and circles (Thellier–Coe method) and squares (565C method) from zircons^41^. We note that the evidence of a field prior to the oldest whole-rock PINT data at 3.45 Ga relies on the single-crystal zircon results, which have been questioned as not deriving from a primary magnetic carrier^42^.

Our results show that a basal magma ocean is likely to have produced a magnetic field (Fig. 3). The magnetic field strength is very similar to that observed in the geologic record of the first few billion years of Earth’s history. Moreover, the magnetic Reynolds number is sufficiently large to allow for a silicate dynamo in the early Earth. Although the basal magma ocean suppresses core convection, the core is likely to have strong horizontal flows that produce toroidal fields from the poloidal field generated by the basal magma ocean, reinforcing the silicate dynamo. The importance of this effect for the silicate dynamo is uncertain. Ziegler and Stegman^19^ suggested as the criterion for magnetic field generation the following dynamo number $D = \left( {R_{\mathrm{m}}R_{\mathrm{{mcore}}}} \right)^{1/2} > 100$, where *R*_mcore_ = 3500 is the magnetic Reynolds number of the core computed assuming that the horizontal flow velocity is similar to that of the core today. According to the dynamo number criterion, the silicate dynamo may operate throughout the Archean (Fig. 3). Alternative velocity scalings also yield *D* > 100 (see Methods). It is likely that the basal magma ocean became more iron-rich as it crystallized and that this iron enrichment increased the electrical conductivity. Our results for *R*_m_ and *D* may therefore be underestimates. We have not attempted a more quantitative treatment of the effects of iron enrichment because liquid-crystal iron partitioning and the magnitude of the effect of iron enrichment on the electrical conductivity of silicate liquid at basal magma ocean conditions are uncertain.

## Discussion

The Earth’s magnetic field may have transitioned from being produced by the basal magma ocean to being produced by the core near the end of the Archean: as the magma ocean cools, heat flow out of the core increases to the point that core convection, and a core dynamo, is possible (Supplementary Fig. 4b). Mechanisms have been proposed by which the core could produce a field earlier, including the presence of radioactive heat producing elements in the core^3,20^ and the precipitation of Mg^4,21^. However, recent experimental studies show that the solubility of K and U in the core is too small to power a dynamo^22,23^. Moreover, a recent reanalysis of experimental data shows that the temperature dependence of Mg precipitation, and thus the power produced, is a factor of six less than originally thought^24^ and may be less than that required to sustain a magnetic field^25^. The field produced by the silicate dynamo may have differed in geometry from the present day field and it may be possible to distinguish these unique characteristics in the geologic record. For example, there is some evidence from modeling magnetic field generation in other planetary bodies, including Uranus and Neptune^26^, that shallow layers, like the basal magma ocean, produce a less dipolar field than the strongly dipolar field of the present Earth. Many super-Earth exoplanets may have extensive magma oceans because of the greater amount of accretional energy stored in larger mass planets^27^, strong stellar irradiation, or the insulation of overlying gaseous envelopes^28^. Silicate dynamos may therefore be common in the universe.

## Methods

### System

Our system consists of 1129 atoms of seven different elements, with relative proportions chosen to closely match the six most abundant oxide components of the bulk silicate Earth (Supplementary Figs. 1 and 2, Supplementary Table 1).

### Molecular dynamics simulations

Our molecular dynamics simulations are based on density functional theory in the PBEsol approximation^29^, combined with the +*U* method^30^ with *U*−*J* = 2.5 eV as in our previous work^10,31,32^. We use the projector augmented wave method^33^, as implemented in VASP^34^. The core radii and number of electrons treated as valence for each element are listed in Supplementary Table 1. Born–Oppenheimer simulations are performed in the canonical ensemble using the Nosé–Hoover thermostat and run for 6–10 ps with 1 fs time step. We perform spin-polarized simulations in which the difference in the number of up-spin and down-spin electrons is fixed to the high spin value (four times the number of Fe atoms) and also non-spin-polarized calculations. We assume thermal equilibrium between ions and electrons via the Mermin functional^35^. Sampling the Brillouin zone at the Gamma point and a basis-set energy cutoff of 500 eV were found to be sufficient to converge energy and pressure to within 2 meV/atom and 0.2 GPa, respectively. Our previous studies^10,11^ indicate that our simulations are well converged with respect to the number of atoms: in the case of SiO_2_, simulations with 96 and 144 atoms did not differ significantly in the value of *σ*, while in the case of (Mg,Fe)O, differences in *σ* between simulations containing 128, 256, and 512 atoms were less than 10%.

### Electronic conductivity and electronic density of states

We compute the electronic conductivity via the Kubo–Greenwood formula^12^ as implemented in VASP from a series of at least 10 uncorrelated snapshots at each volume–temperature condition (Supplementary Fig. 3). The frequency dependent electronic conductivity is

1 $$\sigma _{\mathrm{{el}}}(\omega ) = \frac{{2\pi e^2\hbar ^2}}{{3m^2\omega \Omega }}\mathop {\sum}\limits_{\vec k} {\mathop {\sum}\limits_{i,j} {\left[ {f\left( {\varepsilon _j,\vec k} \right) - f\left( {\varepsilon _i,\vec k} \right)} \right]\left| {\left\langle {\psi _{i,\vec k}} \right\rangle \left| \nabla \right|\left\langle {\psi _{j,\vec k}} \right\rangle } \right|^2\delta (\varepsilon _i - \varepsilon _j - \hbar \omega )} },$$

where the sums are over the Brillouin zone and pairs of states, respectively, *f* is the Fermi occupation, *ψ* is the wavefunction, *ε* is the eigenvalue, *ω* is the frequency, and Ω is the volume of the simulation cell. In our computations, the *δ* function is replaced by a Gaussian of width Δ given by the average spacing between eigenvalues weighted by the corresponding change in the Fermi function^36^. As the behavior of Eq. 1 becomes unphysical for $\hbar \omega \, < \, \Delta$, we find the DC conductivity by linearly extrapolating to zero frequency.

We found that a 1 × 1 × 1 k-point mesh and 7200 electronic bands were sufficient to yield converged values of the electronic conductivity and the electronic density of states by performing calculations at doubled Brillouin zone sampling (2 × 2 × 2 k-point mesh) and a larger number of electronic bands (10,200). We compute the electronic density of states by averaging over 10 snapshots well separated in time from the molecular dynamics trajectories. We use the energy derivative of the Fermi–Dirac function with temperature equal to the ionic temperature to smooth the density of states.

### Ionic conductivity

We compute the ionic part of the electrical conductivity in the DC limit from the electric current autocorrelation function^13^

2 $$J(t) = \mathop {\sum}\limits_{i,j} {z_iz_j\left\langle {\vec u_i(t + t_0) \cdot \vec u_j(t_0)} \right\rangle },$$

as the integral

3 $$\sigma _{\mathrm{{ion}}} = \frac{{e^2}}{{3kT\Omega }}{\int} {J(t){\mathrm{d}}t}.$$

where *z*_*i*_ and $\vec u_i$ are the Bader charge and velocity of ion *i*, respectively, and the angle brackets indicate an average over time origins *t*_*0*_. The Bader charges of the ions computed in our simulation are listed in Supplementary Table 1.

### Thermal evolution of the basal magma ocean

We solve the coupled system of equations ^16^:

4 $$4\pi a^2k\frac{{T_{\mathrm{L}} - T_{\mathrm{M}}}}{\delta } = - (M_{\mathrm{m}}c_{\mathrm{m}} + M_{\mathrm{c}}c_{\mathrm{c}})\frac{{{\mathrm{d}}T_{\mathrm{L}}}}{{{\mathrm{d}}t}} + H(t) - 4\pi a^2\rho \Delta ST_{\mathrm{L}}\frac{{{\mathrm{d}}a}}{{{\mathrm{d}}t}},$$

5 $$T_L = T_A - (T_A - T_B)\xi,$$

6 $$\frac{{{\mathrm{d}}\xi _L}}{{{\mathrm{d}}t}} = - \frac{{3a^2\Delta \xi }}{{a^3 - b^3}}\frac{{{\mathrm{d}}a}}{{{\mathrm{d}}t}},$$

where the first equation expresses the relationship between the time evolution of the outer radius of the basal magma ocean *a*, and its temperature *T*_L_ where *k* is the thermal conductivity, *T*_M_ is the temperature of the overlying solid mantle, *δ* is the thickness of thermal boundary layer at the base of the overlying mantle, *M* and *c* are the mass and isobaric specific heat, respectively of the basal magma ocean (subscript *m*) and core (subscript *c*), *H* is the radioactive heat production, *ρ* is the density of the basal magma ocean, and Δ*S* is the entropy change on freezing. The inner radius of the basal magma ocean *b* is assumed to be the core–mantle boundary. The term on the left-hand side is the total heat flux out of the basal magma ocean. On the right-hand side we have contributions from, respectively, cooling of the basal magma ocean and the core, radioactive heat production, and latent heat of freezing. The second equation expresses the idealized phase diagram defined by a linear dependence of the liquidus temperature on the mass fraction of the dense component *ξ*, where *T*_*A*_ and *T*_*B*_ are the melting temperatures of the end-member light (*A*) and dense (*B*) components. The last equation is a statement of mass balance where Δ*ξ* =  *ξ*_L_ − *ξ*_S_ is the enrichment of the liquid in the dense component. The model does not include inner core growth, nor radioactive heating, nor Mg-exsolution in the core.

We solve the equations with a fourth-order Runge–Kutta method and adopt values of all parameters identical to those assumed in ref. ^16^, except for the entropy of melting, which we take from our previous simulations Δ*S* = 652 J/kg/K^37^ (Supplementary Table 2). The value of *δ* is chosen to yield a present day temperature at the core–mantle boundary of 4000 K.

### Magnetic Reynolds number

We compute the magnetic Reynolds number of the basal magma ocean as

7 $$R_{\mathrm{m}} = \mu _0vL\sigma,$$

where *μ*_0_ is the permeability of free space, *L* is the thickness of the basal magma ocean, *v* is the flow velocity, and *σ* is the electrical conductivity. We take the value of *L* = *a*−*b* from the thermal evolution model (Supplementary Fig. 4). The value of *σ* is the total electrical conductivity computed at the base of the basal magma ocean and the temperature *T*_L_ from the thermal evolution model (Supplementary Fig. 4) and the equations for the temperature dependence of *σ*_el_ and *σ*_ion_ given in the caption to Fig. 1 in the main text.

To estimate the flow velocity, we use the scaling recommended by numerical simulations^17^ from *mixing length theory*

8 $$v = \left( {\frac{{Lq}}{{\rho H_{\mathrm{T}}}}} \right)^{1/3},$$

where *q* is the total heat flow out of top of the basal magma ocean (Supplementary Fig. 4b), *ρ* is the density of the magma ocean, and *H*_T_ is the thermal scale height, which we find from our previous simulation results^37^ to be *H*_T_ = 7622 km. Following^19^, we also examine alternative scalings for the velocity, including those based on a balance between Coriolis, inertial, and Archimedean (buoyancy) forces: *CIA*

9 $$v = \left( {\frac{q}{{\rho H_{\mathrm{T}}}}} \right)^{2/5}\left( {\frac{L}{\Omega }} \right)^{1/5}$$

and from a balance between the buoyancy and Lorentz forces: *MAC*

10 $$v = \left( {\frac{q}{{\Omega \rho H_{\mathrm{T}}}}} \right)^{1/2},$$

where Ω is the rotation rate.

We also estimate the possible effects of coupling of the magnetic field produced by the basal magma ocean with the underlying, non-convecting core. Although the basal magma ocean suppresses core convection, the core is likely to have strong horizontal flows that generate toroidal fields from the poloidal field produced by the basal magma ocean, reinforcing the silicate dynamo. Ziegler and Stegman^19^ suggested as the criterion for magnetic field generation the following dynamo number *D*: the geometric mean of the magnetic Reynolds numbers of the basal magma ocean and the core

11 $$D = \left( {R_{\mathrm{m}}R_{m{\mathrm{{core}}}}} \right)^{1/2},$$

where *R*_mcore_ = 3500 is the magnetic Reynolds number of the core computed assuming that the horizontal flow velocity is similar to that of the core today, and *D* > 100 in order to produce a magnetic field. Following Ziegler and Stegman, we have estimated *R*_mcore_ by assuming that the horizontal flow velocity is similar to that of the core today (*v* = 5 × 10^−4^ m/s)^38^, the conductivity, *σ* = 1.5 × 10^6^ S/m^39^, and *L* = 3500 km.

## Supplementary information

Supplementary Information

## Data Availability

Raw simulation results are available from the corresponding author on request.

## Code availability

VASP is widely used proprietary software.
