# Supplementary material for: A silicate dynamo in the early Earth
Source: Nat Commun. 2020 Feb 25;11:935. doi: 10.1038/s41467-020-14773-4 (PMC7042358; doi:10.1038/s41467-020-14773-4)
Supplement: Supplementary file 1 — Supplementary Information [file 41467_2020_14773_MOESM1_ESM.pdf]

Supplementary Information for

A Silicate Dynamo in the Early Earth

by

Lars Stixrude, Roberto Scipioni, and Michael P. Desjarlais

Supplementary Tables

Supplementary Table 1. Composition of the simulated system given as the number of atoms in the periodically repeated simulation cell (Number) and as the mass fraction of conventional oxides (wt. %) compared with the depleted mid-ocean-ridge-basalt mantle (DMM) composition of Workman and Hart [1]. Also shown are the core radii and number of valence electrons of the PAW potentials used for each element, and the Bader charge computed for each ion [2].

Element	Number	Oxides wt. %	DMM wt. %	Radius (Bohr)	Valence Electrons	Bader Charge
Si	178	44.93	44.93	1.90	4	2.77
Mg	229	38.78	38.82	2.00	8	1.75
Fe	28	8.45	8.56	2.20	14	0.80
Ca	14	3.30	3.18	3.00	8	1.39
Al	20	4.28	4.37	1.90	3	2.34
Na	2	0.26	0.13	2.20	7	0.81
O	658			1.52	6	-1.48

Supplementary Table 2. Values of parameters used in the thermal evolution model.

Parameter		Value
k	Thermal conductivity	8 W m^{-1} K^{-1}
δ	Boundary layer thickness	87 km
T_M	Mantle temperature	2730 K
c_m	Basal Magma ocean specific heat	1000 J kg^{-1} K^{-1}
c_c	Core specific heat	860 J kg^{-1} K^{-1}
M_c	Core mass	2x10^{24} kg
ρ	Basal magma ocean density	5500 kg m^{-3}
ΔS	Entropy of melting	652 J kg^{-1} K^{-1}
$\Delta\xi$	Mass fraction change on freezing	0.088
T_A	Component A melting temperature	5500 K
T_B	Component B melting temperature	3500 K
b	Core radius	3480 km
Ω	Rotational period	24 hours

Supplementary Figures

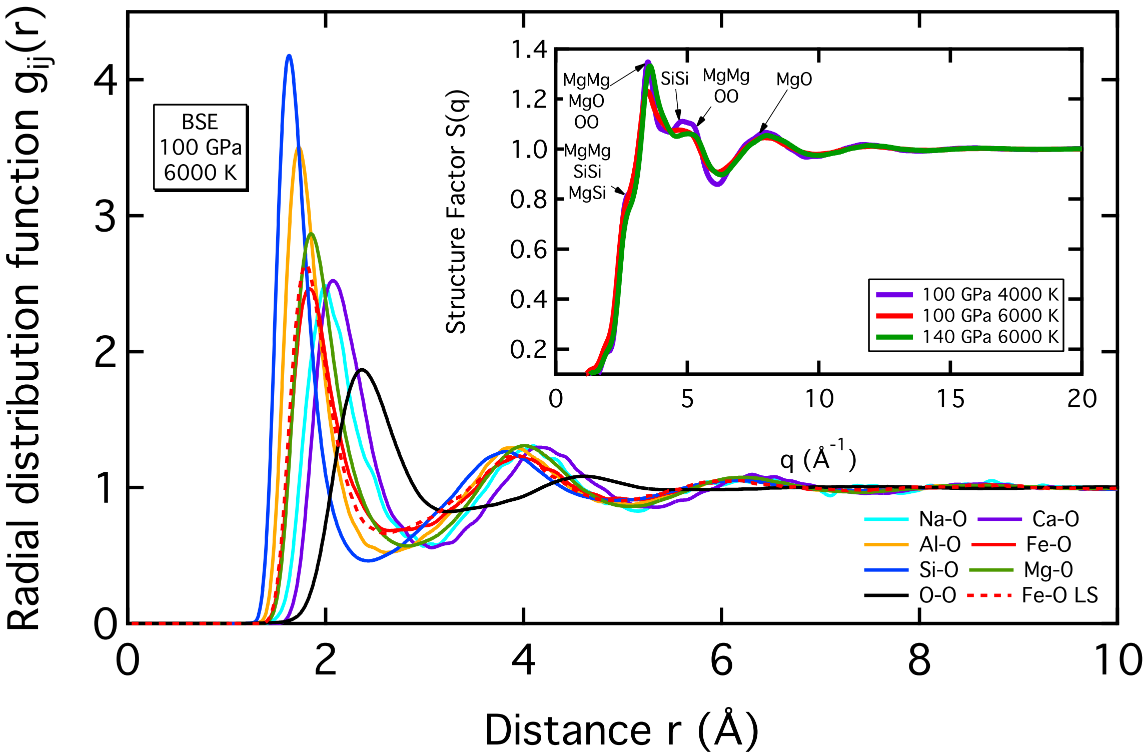

Supplementary Figure 1. Structure of the bulk silicate Earth composition liquid as represented by the oxygen partial radial distributions functions from a fully spin-polarized simulation (solid lines). For comparison, we also show, in the case of Fe-O, the result for a non-spin-polarized (LS) simulation at the same pressure and temperature (dashed line), which produces shorter Fe-O distances. The inset shows the x-ray structure factor computed by convolving the Fourier transform of the radial distribution function with the x-ray scattering factors of the ions. Labels indicate the dominant contributions to the peaks and shoulders.

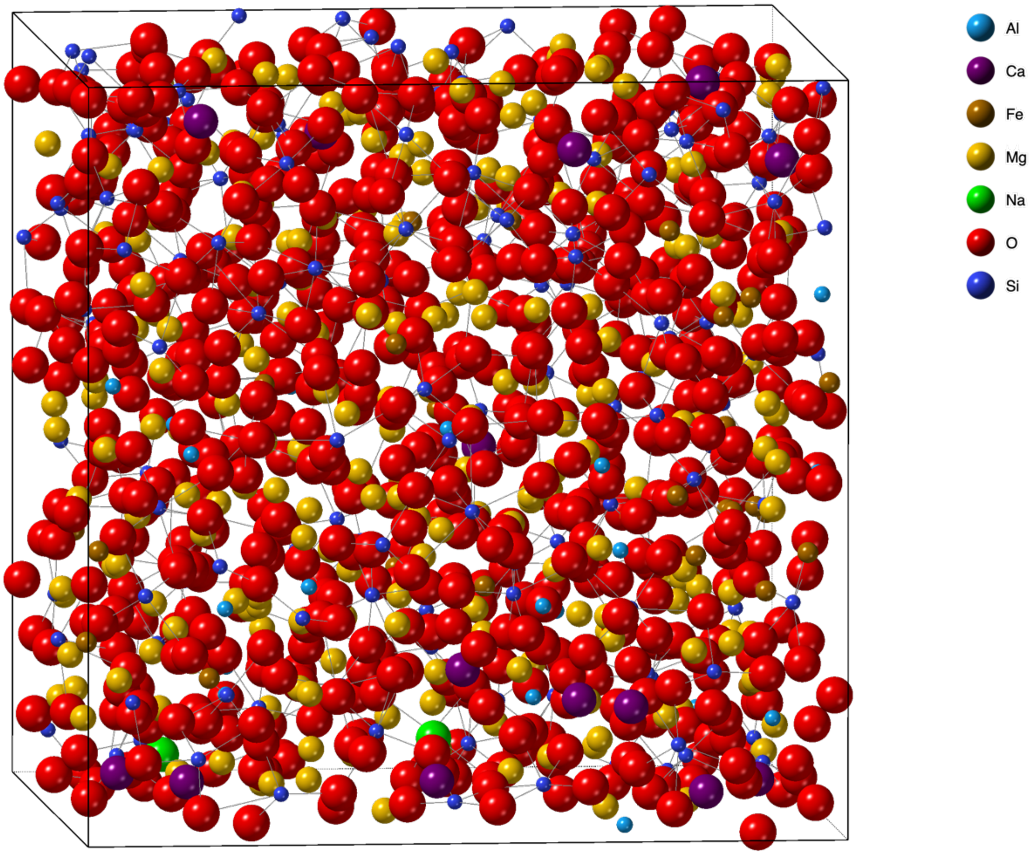

Supplementary Figure 2. Snapshot from a simulation at 100 GPa and 4000 K.

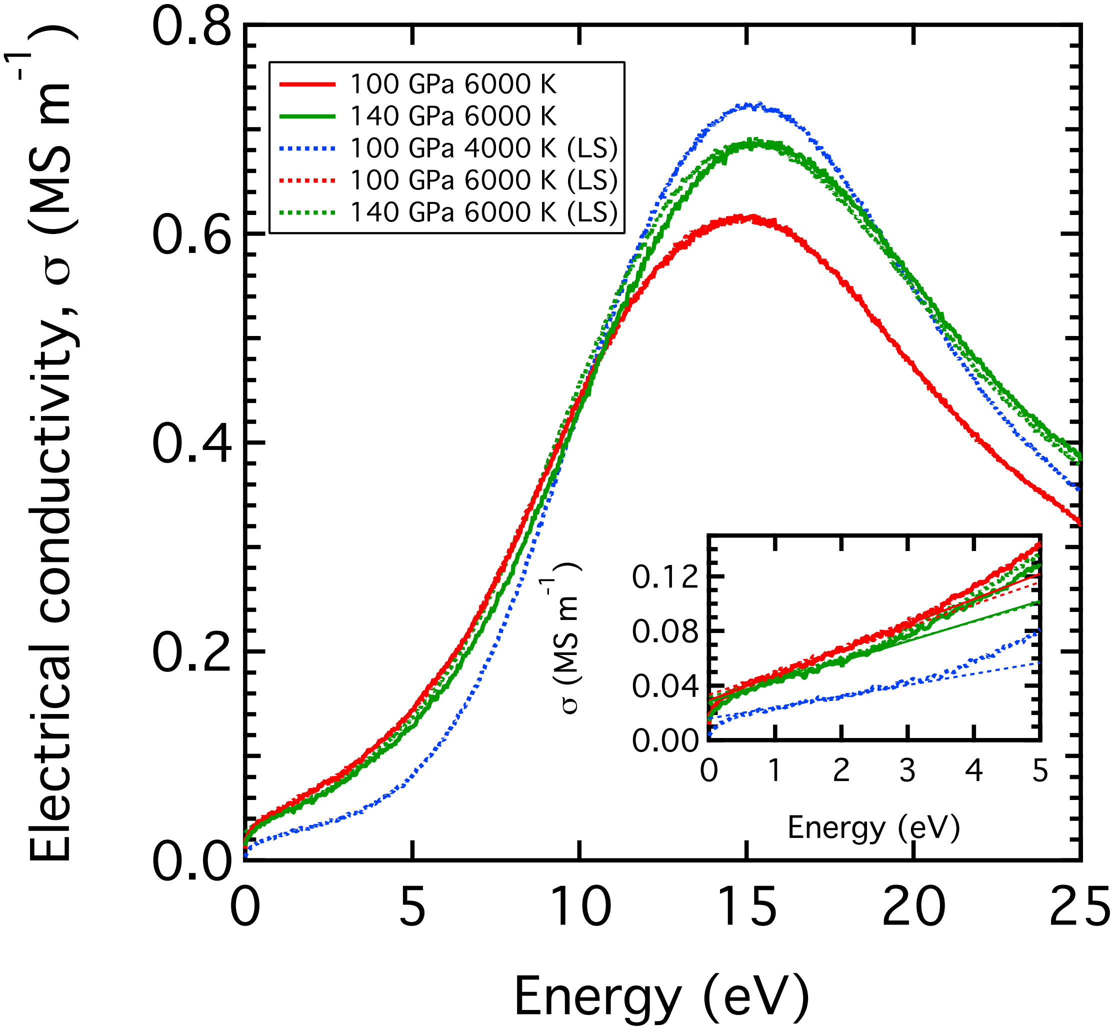

Supplementary Figure 3. Electronic conductivity as a function of energy at the pressure-temperature conditions indicated from spin polarized (solid) and non-spin polarized (dashed) calculations. We find the zero frequency (DC) value of the electronic conductivity as the intercept of the best fit line to results between 1 and 2 eV as shown in the inset.

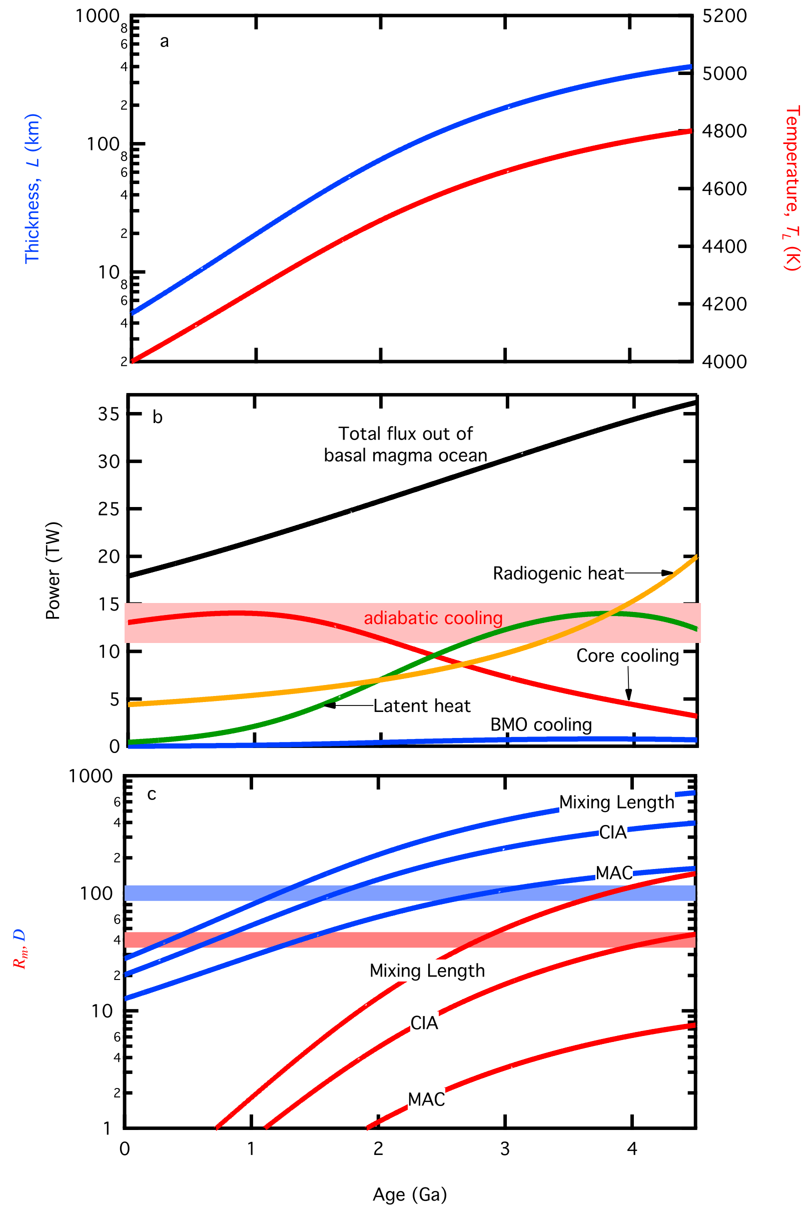

Supplementary Figure 4. Thermal evolution of the basal magma ocean. **a** The thickness (blue) and temperature (red) as a function of time. **b** The total heat flux out of the basal magma ocean (black) and the contributions to this total: cooling of the basal magma ocean (blue), cooling of the core (red), radiogenic heat (orange), and latent heat of freezing (green). The pink band shows the heat flow conducted down the core adiabat according to the values of the thermal conductivity of the core predicted by[3] (top of band, thermal conductivity at the top of the core k_{core}=100 W/m/K) and[4] (bottom of band, k_{core} =77 W/m/K). The pink band indicates the minimum core cooling required for thermal convection of the outer core. **c** Values of the magnetic Reynolds number computed from the thermal evolution model and velocity scaling laws indicated (red lines, Eqs. 0.7-0.10) and the dynamo number (blue lines, Eq. 0.11) as compared with their critical values for dynamo generation (thick horizontal lines).

SUPPLEMENTARY REFERENCES

1. Workman, R. K. & Hart, S. R. Major and trace element composition of the depleted MORB mantle (DMM). *Earth And Planetary Science Letters* **231**, 53-72 (2005).
2. Tang, W., Sanville, E. & Henkelman, G. A grid-based Bader analysis algorithm without lattice bias. *Journal of Physics-Condensed Matter* **21**, 7, doi:10.1088/0953-8984/21/8/084204 (2009).
3. Pozzo, M., Davies, C., Gubbins, D. & Alfé, D. Thermal and electrical conductivity of iron at Earth's core conditions. *Nature* **485**, 355-U399 (2012).
4. Xu, J. Q. *et al.* Thermal Conductivity and Electrical Resistivity of Solid Iron at Earth's Core Conditions from First Principles. *Physical Review Letters* **121**, 6, doi:10.1103/PhysRevLett.121.096601 (2018).
